# Midterm functional outcome after laparoscopic ventral rectopexy for external rectal prolapse

**DOI:** 10.1111/ases.12701

**Published:** 2019-03-28

**Authors:** Akira Tsunoda, Tomoko Takahashi, Satoshi Matsuda, Naoki Oka, Hiroshi Kusanagi

**Affiliations:** ^1^ Department of Gastroenterological Surgery, Kameda Medical Center Kamogawa Japan; ^2^ Department of Pediatric Surgery, Kameda Medical Center Kamogawa Japan

**Keywords:** external rectal prolapse, laparoscopic ventral rectopexy, midterm results

## Abstract

**Introduction:**

Although long‐term crude outcomes of laparoscopic ventral rectopexy for external rectal prolapse (ERP) have been documented, repetitive functional and quality of life (QOL) assessments are scarce. This study assessed midterm annual functional results and QOL after laparoscopic ventral rectopexy for ERP.

**Methods:**

This study consisted of 58 patients and was a retrospective analysis of prospectively collected data. The Fecal Incontinence Severity Index, the Constipation Scoring System, and QOL instruments (ie 36‐item Short‐Form Health Survey and Fecal Incontinence Quality of Life scale) were administered before and after operation.

**Results:**

There was no mortality or major morbidity. After a median follow‐up of 49 months (6‐92 months), recurrence of ERP was noted in one patient (2%). There were no mesh‐related complications. The median Fecal Incontinence Severity Index score was significantly reduced at 3 months (34 [10‐61] vs 12 [0‐50], *P* < 0.0001) and remained significantly reduced for 5 years. The median Constipation Scoring System score was significantly reduced at 3 months (14 [9‐20] vs 7 [0‐16], *P* < 0.0001) and remained significantly reduced for 4 years. No patients developed new‐onset constipation. All of the Fecal Incontinence Quality of Life scales significantly improved overtime for 4 years. All of the 36‐item Short‐Form Health Survey scales were significantly improved at 3 and 6 months, but none of the scales significantly improved after 2 years.

**Conclusion:**

Laparoscopic ventral rectopexy for ERP was associated with low morbidity, low recurrence, and a midterm improvement in function and fecal incontinence‐specific QOL.

## 1 INTRODUCTION

The ideal surgical treatment for external rectal prolapse (ERP) should correct the related anatomical abnormalities and derived symptoms, which range from fecal incontinence (FI) to obstructed defecation (OD). Abdominal posterior rectopexy is preferable to a perineal procedure because it has a much lower incidence of long‐term recurrence and offers better recovery of continence.^1^ However, such abdominal procedures often provide poor resolution and induce new‐onset constipation. The cause of this postoperative constipation remains unclear, but nerve injuries during extensive posterior rectal mobilization leading to complete denervation may be involved.^2^

In the past decade, nerve‐sparing laparoscopic ventral rectopexy (LVR) has been recognized as a treatment for ERP. There is increasing evidence that LVR not only controls the prolapse but also improves the associated symptoms.^2^, ^3^, ^4^ This anterior approach limits rectal mobilization without lateral dissection, reducing the incidence of postoperative constipation, as compared to posterior rectopexy.^2^, ^3^, ^4^, ^5^ Although long‐term crude outcomes of LVR for ERP have been documented, limited information on repetitive functional results is available.^6^, ^7^, ^8^ Additionally, only one study has evaluated the long‐term impact of LVR on health‐related quality of life (QOL); it employed instruments on global gastrointestinal symptoms, but major symptoms of FI resulting from ERP were not assessed.^8^ This study aimed to assess midterm annual functional results and QOL after LVR by using both generic and FI‐specific instruments in a consecutive series of patients with ERP.

## 2 MATERIALS AND METHODS

Data for all 58 patients who underwent LVR for ERP between September 2011 and April 2015 were prospectively entered into a pelvic floor database. This study group included the first 31 cases from our previous study.^4^

The diagnosis of ERP was made clinically and, where this was not possible, on evacuation proctography. No patient underwent a colonic transit study.

After the risk of mesh‐related complications was explained, informed consent was obtained from each patient. This study was approved by the Ethical Committee of Kameda Medical Center (approval no.18‐036). Information on the study protocol was made public, and patients were ensured that they could withdraw consent. However, no patients or their relatives subsequently refused to participate the study.

### 2.1 Operative technique

Surgical procedures were performed as previously described.^4^, ^9^ During the series, polypropylene mesh was secured to the sacral promontory by using an endofascial stapler in the first 46 cases and a ProTack device (Autosuture, Tyco Healthcare, Mansfield, MA) in the remaining 12 cases.

### 2.2 Evacuation proctography

A standardized proctography technique was used. Proctograms were evaluated according to the criteria proposed by Shorvon et al.^10^ Briefly, ERP was diagnosed when the full thickness of the rectum protruded through the anal orifice. Based on the images taken during maximal straining defecation, rectoanal intussusception (RAI) was diagnosed when the apex of the rectal intussusception impinged on the internal anal orifice or was intra‐anal. Rectorectal intussusception (RRI) was diagnosed if the apex remained intrarectal and did not impinge on the internal anal orifice. The presence of rectocele was classified as grade l (<2 cm in depth), grade 2 (2‐4 cm in depth), or grade 3 (>4 cm in depth).^11^ Enterocele or sigmoidocele was diagnosed when the extension of the loop of the bowel was located between vagina and rectum. Pelvic floor descent during defecation was estimated by the degree of the anorectal junction in relation to the inferior margin of the ischial tuberosity.

### 2.3 Incontinence and constipation

The Fecal Incontinence Severity Index (FISI) score was used to quantify the degree of incontinence on a scale of 0‐61, with 61 indicating total incontinence.^12^ The Constipation Scoring System (CSS) score was used to quantify constipation on a scale of 0‐30 points, with a higher score indicating worse constipation.^13^

### 2.4 Quality of life

The validated Japanese version of 36‐item Short Form Health Survey (SF‐36) was used to assess generic QOL based on eight domains: (i) physical functioning; (ii) physical role functioning; (iii) bodily pain; (iv) general health; (v) vitality; (vi) social functioning; (vii) emotional role functioning; and (viii) mental health. The SF‐36 was scored on a scale of 0 (worst QOL) to 100 (perfect QOL).^14^ The validated Japanese version of Fecal Incontinence Quality of Life scale (FIQL) measured QOL in the patients with FI.^15^ It assessed 29 items of four scales (lifestyle, coping/behavior, depression/self‐perception, and embarrassment), with a higher score reflecting better QOL.

### 2.5 Follow‐up

Patients were followed‐up 3, 6, and 12 months and annually thereafter for up to 5 years. The FISI, CSS, SF‐36, and FIQL were completed at each follow‐up visit. Nursing staff distributed the self‐administered questionnaires to patients in the outpatient clinic. If patients did not have a checkup, they were asked to report their FISI and CSS scores by phone and to return the QOL questionnaires by mail. Those who indicated that they had a feeling of prolapse were examined in the clinic. In patients in whom reintervention for recurrence or new‐onset RAI was indicated, the functional outcome was noted at the last follow‐up before the intervention.

### 2.6 Statistical analysis

Quantitative data are expressed as the median and range. Analysis was performed with the Mann‐Whitney *U* test for unpaired data. The Wilcoxon signed‐rank test for paired data was used to compare pairs of functional or QOL scores. Categorical variables were compared by using the *χ* ^2^ test or Fisher's exact test. The data were analyzed with SPSS version 11.0 for Windows (SPSS Japan Institute, Tokyo, Japan). *P* < 0.05 was considered to indicate statistical significance.

## 3 RESULTS

The median age of patients was 80 years (40‐94 years), and 52 patients (90%) were women. Five patients were classified as ASA grade 1, 44 as ASA grade 2, and 9 as ASA grade 3. The nine patients classified as ASA grade 3 had been diagnosed with cardiac disease (n = 6), chronic renal failure (n = 1), chronic pulmonary disease (n = 1), and diabetes mellitus requiring insulin therapy (n = 1). The median interval from the operation to the end of June 2018 was 60 months (36‐92 months), and the median duration of follow‐up was 49 months (6‐92 months) (Table 1). During the follow‐up period, 12 patients (21%) died of causes unrelated to LVR at a median of 27 months (6‐59 months) after surgery without recurrence. Anorectal function and QOL could not be assessed in 11 patients who had senile dementia or schizophrenia.

**Table 1:** Patient characteristics

Age (years), median (range)	80 (40‐94)
Sex (n)	
Male	6
Female	52
ASA Physical Status (n)	
1	5
2	44
3	9
Previous surgery for external rectal prolapse (n)	
Delorme's procedure	4
Repeated Delorme's procedure	4
Altemeier's procedure	1
Repeated Altemeier's procedure	1
Gant‐Miwa procedure	1
Senile dementia or schizophrenia (n)	11
Follow‐up period (months), median (range)	49 (7‐92)

There was no conversion to open surgery or surgical reintervention during the primary surgery admission. Eight patients with pelvic organ prolapse underwent LVR and sacrocolpopexy. The median length of the postoperative hospital stay was 1 day.^1^, ^2^, ^3^, ^4^, ^5^, ^6^, ^7^, ^8^ There was no postoperative mortality or major morbidity. Six patients had grade I complications according to the Clavien–Dindo classification. No patients were readmitted for medical or surgical complications.

### 3.1 Evacuation proctography

Preoperative evaluation was performed in 34 patients. The findings indicated that in addition to ERP, five women had enterocele, and three had sigmoidocele. Six months after surgery, proctography was performed in 39 patients, including the 34 patients evaluated preoperatively. ERP was not present in any of the patients, although 11 had RAI and four had RRI. Enterocele disappeared in all five patients, but the site of herniation of the small bowel moved posteriorly to a point along the rectum in two patients. Sigmoidocele disappeared in two patients, and the mesh had detached in one. Pelvic floor descent was not significantly reduced postoperatively (preoperatively vs postoperatively: 21.1 [−8.8‐51.3] vs 18.9 [6.4‐46.1] mm, *P* = 0.51) (Table 2).

**Table 2:** Evacuation proctography findings

	Preoperative (n = 34)	At 6 months (n = 39)
External rectal prolapse (n)	34	0
Rectoanal intussusception (n)	0	11
Rectorectal intussusception (n)	0	4
Enterocele (n)	5	0
Sigmoidocele (n)	3	1
Pararectal hernia^a^ (n)	0	2
Pelvic floor descent (mm)^b^, median (range)	21.1 (−8.8‐51.3)	18.9 (6.4‐46.1)*

a Enterocele was eliminated in all five patients, but the site of herniation of the small bowel moved posteriorly to a point along the rectum in two patients.

b The extent of the anorectal junction relative to the inferior margin of the ischial tuberosity during defecation.

* *P* = 0.51, versus preoperatively (Wilcoxon signed‐rank test).

### 3.2 Recurrence and further procedures

Forty‐eight patients (83%) were followed up for at least 3 years to monitor for recurrent ERP. At the end of the follow‐up period, ERP had recurred in only one patient (2%), who had undergone Delorme's operation 12 months after the initial surgery. Of the 11 patients with new‐onset RAI, 4 underwent reoperation because FI and/or OD symptoms had not improved. In two of these patients, mesh had detached from the sacrum, and re‐LVR with another mesh fixed at the sacral promontory was performed 17 and 31 months after the primary surgery, respectively. After reoperation, FI symptoms improved, but OD symptoms persisted in both cases. The other two patients underwent Delorme's transanal excision for mucosal prolapse 10 and 41 months after the initial surgery, respectively. There were no further mesh‐related complications.

### 3.3 Fecal incontinence

Of the 47 assessable patients following the exclusion of 11 patients with senile dementia or schizophrenia from the subjects, 46 patients (98%) presented with FI preoperatively. FISI score was significantly reduced at 3 months (preoperatively vs postoperatively: 34 [10‐61] vs 12 [0‐50], *P* < 0.0001) and remained significantly reduced for 5 years. However, the number of evaluated patients decreased year by year. At 3 months, 67% of patients had a reduction of at least 50% in their FISI score, and this was true of a greater percentage of patients thereafter (Table 3).

**Table 3:** Fecal Incontinence Severity Index scores

Time	Evaluated patients (n)	Score, median (range)	Patients with significant improvement,^a^ n (%)	*P*‐value^b^
Preoperative	46	34.0 (10.0‐61.0)	—	—
3 months	46	12.0 (0.0‐50.0)	30 (67)	<0.0001
6 months	42	12.0 (0.0‐41.0)	32 (76)	<0.0001
12 months	42	9.5 (0.0‐33.0)	26 (77)	<0.0001
2 years	21	4.0 (0.0‐43.0)	17 (81)	<0.0001
3 years	20	8.0 (0.0‐37.0)	17 (85)	<0.0001
4 years	13	10.0 (0.0‐32.0)	10 (77)	0.002
5 years	10	7.0 (0.0‐21.0)	9 (90)	0.005

a Reduction of at least 50% in Fecal Incontinence Severity Index score after laparoscopic ventral rectopexy.

b Versus preoperative (Wilcoxon signed‐rank test).

### 3.4 Constipation

Constipation was not assessable in 11 patients with senile dementia or schizophrenia. Twenty‐three of the remaining 47 assessable patients presented with constipation preoperatively. CSS score was significantly reduced at 3 months (preoperatively vs postoperatively: 14 [9‐20] vs 7 [0‐16], *P* < 0.0001) and remained significantly reduced for 4 years. However, the number of evaluated patients decreased year by year. At 3 months, 52% of patients had a reduction of at least 50% in their CSS score, and this was true of a greater percentage of patients thereafter (Table 4). The 24 patients without preoperative constipation did not develop new‐onset constipation.

**Table 4:** Constipation Scoring System scores

Time	Evaluated patients (n)	Score, median (range)	Patients with significant improvement,^a^ n (%)	*P*‐value^b^
Preoperative	23	14.0 (9.0‐20.0)	—	—
3 months	23	7.0 (0.0‐16.0)	12 (52)	<0.0001
6 months	22	6.5 (1.0‐15.0)	12 (55)	<0.0001
12 months	16	5.5 (1.0‐12.0)	12 (75)	0.001
2 years	8	3.5 (1.0‐16.0)	6 (75)	0.03
3 years	8	4.5 (2.0‐13.0)	6 (75)	0.01
4 years	6	3.5 (1.0‐17.0)	5 (83)	0.04
5 years	4	2.0 (0.0‐13.0)	3 (75)	0.11

a Reduction of at least 50% in Constipation Scoring System score after laparoscopic ventral rectopexy.

b Versus preoperative (Wilcoxon signed‐rank test).

### 3.5 Quality of life

**Figure 1:**
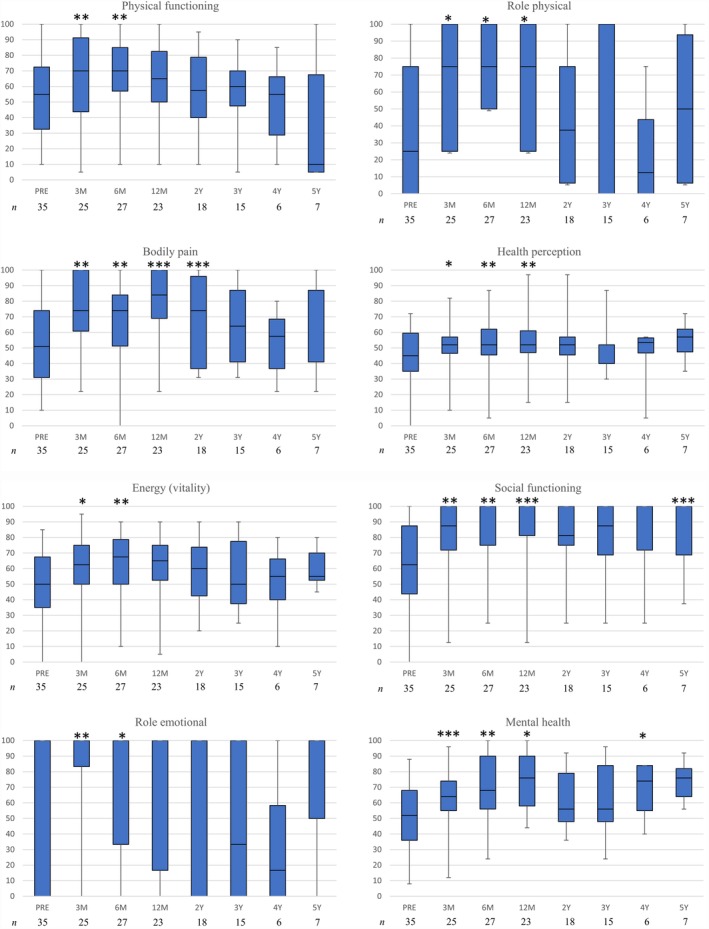
Scores from the 36‐item Short Form Health Survey in patients with external rectal prolapse and rectoanal intussusception. The boxes show median values with upper and lower quartiles. The vertical lines extend from the minimum to the maximum values. Abbreviations: *n,* number of patients who responded to each questionnaire. Preoperative scores were compared with postoperative scores by using the Wilcoxon signed‐rank test. *P < 0.05. **P < 0.01. ***P < 0.0001

Of the 47 assessable patients, 35 patients submitted QOL questionnaires preoperatively; the remaining 12 patients did not report or receive the questionnaires. The number of patients who submitted QOL questionnaires at each postoperative follow‐up was not consistent and decreased with time. All of the SF‐36 scales were significantly improved at 3 and 6 months. The scores of three domains (physical functioning, vitality, emotional role functioning) did not remain significantly improved at 12 months and thereafter. The scores of the four domains (physical role functioning, general health, social functioning, and mental health) did not remain significantly increased at 2 years and thereafter. Bodily pain domain scores remained significantly increased for 2 years (Figure 1).

**Figure 2:**
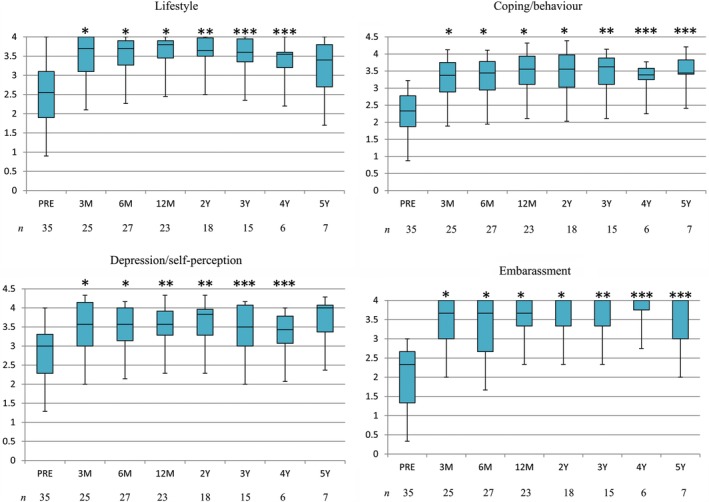
Fecal Incontinence Quality of Life scores in patients with external rectal prolapse and rectoanal intussusception. The boxes show median values with upper and lower quartiles. The vertical lines extend from the minimum to the maximum values. Abbreviations: *n,* number of patients who responded to each questionnaire. Preoperative scores were compared with postoperative scores by using the Wilcoxon signed‐rank test. *P < 0.0001. ^**^ *P* < 0.01. ***P < 0.05

All of the FIQL scales were significantly improved over time by LVR. The scores were significantly increased at 3 months. Lifestyle and depression/self‐perception scores remained significantly increased for 4 years, and coping/behavior and embarrassment scores did so for 5 years (Figure 2).

## 4 DISCUSSION

This study assessed midterm functional results and QOL after LVR for ERP. After a median follow‐up period of 49 months, the recurrence rate was low (2%). During follow‐up, incontinence improved in more than two‐third of patients, and constipation improved in more than half of patients. No patients developed new‐onset constipation. FIQL was better postoperatively and continued to improve significantly for at least 4 years. These data confirm that LVR is an effective and reliable surgical treatment for ERP.

The current literature on long‐term follow‐up after LVR for ERP is limited, but several studies have reported significant postoperative improvements in FI and OD symptoms in patients with ERP.^2^, ^6^, ^7^, ^8^ However, these studies did not measure functional outcomes every year or report outcomes after 2 to 5 years. Consten et al reported that in 242 patients who underwent LVR for ERP,^7^ FI symptoms had improved in 63% of patients (62/98) and OD symptoms in 60% of patients (50/82) at a median follow‐up of 34 months. Although our assessment of symptom improvement differed from that of other authors—with significant improvement defined as a reduction of at least 50% in FISI or CSS scores in the present study—but postoperative continence was satisfactory and significantly improved by more than 80% at 2 years and thereafter. Likewise, constipation significantly improved by more than 70% at 2 years and thereafter. The mechanisms of postoperative improvement in OD symptoms are unclear but may be related to autonomic nerve‐sparing surgery.^2^ LVR does not require posterior and lateral rectal mobilization, thus avoiding the risk of sympathetic nerve damage. However, caution is necessary when interpreting the outcomes because constipation may not improve postoperatively in those who develop new‐onset RAI on postoperative proctography.^4^ Indeed, two of the five patients with new‐onset RAI who had preoperative OD symptoms and did not undergo a reintervention after LVR died of a cause unrelated to LVR in this study; they were not followed at 2 years and thereafter. Although no patient developed new‐onset constipation in this study, previous studies reported incidences of 1.4% and 2.4%.^7^, ^16^

This study provided prospective data about LVR's impact on global QOL and FI‐specific QOL based on responses to the SF‐36 and FIQL, respectively. Long‐term improvement in QOL after LVR was reported by a previous study that used the gastrointestinal quality of life form (GIQLI).^8^ However, generic QOL instruments such as the GIQLI are unable to distinguish differences in clinical severity between individuals. Therefore, symptom‐specific instruments such as the FIQL should be used to evaluate the symptom severity. Our previous study showed that all of the FIQL scales significantly improved after LVR in patients with ERP for the first year.^17^ This study found that these improvements remained significant for 4 years. Meanwhile, none of the SF‐36 scales was significantly improved after 2 years, but all of the SF‐36 scales were significantly improved at 3 and 6 months. This may be because the number of evaluated patients began to decrease at 2 years, so the findings did not reach statistical significance. Another possible reason may be that our patients were more elderly. The median age of the 35 patients who preoperatively submitted a SF‐36 was 78 years (40‐89 years), which was more than 10 years older than those in the previous study about LVR's impact on generic QOL.^8^ Over time, elderly patients are increasingly at risk of becoming prefrail or frail year. A recent systemic review demonstrated that frailty may be associated with poorer generic QOL.^18^

The 2% recurrence rate observed in this study after a median follow‐up of 49 months compares favorably to previous studies, which reported recurrence rates ranging from 3% to 5%.^2^, ^6^, ^7^ Randall et al reported recurrences in 4 of 120 patients (3%) in a study with a median follow‐up of 44 months,^6^ and Consten et al reported recurrences in 13 of 242 patients (5.4%) in a study with a median follow‐up of 34 months.^7^ The patient with recurrent prolapse in the present study was the same one documented in our initial series of 31 cases,^4^ suggesting that a correction of anatomical defect was maintained for many years after LVR in most cases.

Previous studies have reported that LVR is an effective treatment for enterocele.^4^, ^9^ The mesh elevates the pouch of Douglas and corrects a concomitant enterocele and sigmoidocele. In this study, these effects of LVR were supported by postoperative proctography, which showed that enterocele disappeared and that sigmoidocele was corrected as long as the mesh remained attached. However, it is uncertain whether such anatomical correction remains for years after LVR. Four patients with new‐onset RRI experienced improvement in their presenting symptoms. An earlier proctographic study found that patients with RAI were more likely to experience symptoms of OD and FI than patients with RRI.^19^

The review of postoperative morbidity showed that LVR is safe and can be performed on elderly patients with minimal complications, which was consistent with previous studies.^20^, ^21^ Our patients were 10 to 20 years older than those in previous larger studies and did not have major complications or require readmission.^6^, ^7^, ^21^, ^22^ Gultekin et al showed that major complications did not significantly differ between patients who were <80 and ≥80 years old,^23^ but the elderly group had more comorbidities such as cardiac disease. Wijffels et al reported that LVR is safe for perineal procedures in elderly patients who are very frail.^20^

Concerns about LVR relate to potential mesh‐related infection or mesh erosion into the rectum or vagina.^21^, ^24^ Evans et al reported 2% of patients (45/2203) developed mesh erosion.^21^ No patient in our series experienced these complications, but two patients with new‐onset RAI required reoperation because of mesh detachment. Full‐thickness recurrence was not evident on postoperative proctography.

This study was limited by the small sample size, decreasing number of evaluated patients over time, and lack of a control group.

In conclusion, this study confirmed that LVR is an effective treatment for ERP, with low morbidity and a low recurrence rate. This minimally invasive procedure showed a midterm improvement of function, particularly with regard to incontinence, as supported by the symptomatic QOL instruments.
